# Loot

**Published:** 1867

**Authors:** 


### LOOT.

*Cholera.*—Alexander Charles Macleod (with many suffixes,) tells us that:

"Cholera is an imponderable matter, or condition of matter; tellurial in its origin; existent in and with the atmosphere, but forming no component part of it, being of it as independent as are the rays of light; attracted by some other matter or condition of matter, existent in and peculiar to the human body. Where a great space, as a continent or arm of the sea, intervenes, the earth itself may become the conducting medium."

With which satisfactory explanation we must fain be content for the present, meanwhile revolving in our own minds the mediæval problem: "Whether a Chimæra ruminating in a vacuum doth devour second intentions?"

*Vesical Calculus.*—W. D. Winer, M. D., late surgeon of Col. Mulligan's Regiment, U. S. A., has recently shown us a very ingenious arrangement by which he hopes or, rather, *expects*, to be able to surround a calculus, whilst still within the bladder, with an artificial cyst into which can be injected solutions of appropriate strength and character to dissolve the stone. The apparatus seems scarcely as complicated as many which have been suggested and employed in lithotrity. The main practical trouble seems to be in securing a membrane of the required tenuity which at the same time will be tenacious enough to resist the very slight force required.

*Clitoridectomy.*—The JOURNAL, rejoicing in every good word and work, also is pleased now and then at chronicling disuse of the bad and worse. Among these counted worse, is the feminine mutilation named at the head of this paragraph. No less an authority than CHARLES WEST says:—"Whilst I believe the removal of the clitoris in cases of hysteria, epilepsy, insanity, and other nervous diseases of women, to be a proceeding theoretically based on erroneous physiology, and practically followed by no such results as to warrant its frequent performance, I regard it as completely unjustifiable when done for the alleged relief of dysuria, or of painful defecation, for the cure of amenorrhœa, or the mitigation of the symptoms of uterine misplacement or disease." All which we steadfastly believe. Now, let some one, equally high in rank in the Medical Republic, raise his voice against the womb-burning, slitting, and impaling which everywhere with equal *un*philosophy prevails, and the JOURNAL will rejoice yet again with joy, perhaps unspeakable, but which nevertheless shall be written.

*Poisoning by Strychnine.*—Dr. John Bartlett, formerly of Chicago, strongly recommends common salt as a curative of strychnine poisoning. He reports as many as twenty experiments on dogs, in which violent symptoms, following large doses of strychnia, ceased after emesis, induced by drenching the animals with water holding in solution several handfuls of salt.

*Milk-Sickness.*—Quite a spirited controversy, through the local press, is going on in Clay County, Ill., on the permanent question: Whether there be such an entity as "Milk Sick," as distinct from a mere variety of Bilious Remittent, and "if so be there be," whether any one has seen it this side of "the next county," where it always has been the editorial fortune to find it located, or rather *dis*located. The JOURNAL is inclined to put "Milk Sick" in the same category with wounds from poisoned bullets of old time, poisoned wells in the late war, and demoniac possession of antiquity. Medical ontologists are rapidly disappearing in these latter days.

*Elimination in Cholera.*—The American Journal of Medical

Sciences endorses fully the conclusions of Prof. LIONEL BEALE, viz.:—[Italics ours.]

1. That the gland-cell is not, *as a general rule* destroyed when it secretes.

2. That the poisons "eliminated" by the skin and kidneys are *probably* in a state of solution.

3. That the poisons of contagious diseases *are not soluble*, but consist of living germs which move of themselves, but which cannot be "eliminated" from the blood by epithelial or other cells.

4. That so far from their being any evidence of the epithelial cells eliminating contagious poisons, the living particles of the latter interfere with the action of the cells, and many cells are destroyed by them.

5. That the functions of the columnar epithelial cells is to draw substances from the intestine and pass them on towards the blood, and that therefore it is *almost improbable* that these cells should take part in "eliminating" anything whatever from the blood.

To which propositions, supported by *ifs* and *probables*, we are called upon to sacrifice the only clear and scientific theory of cholera which has yet been given. This JOURNAL demurs. The *third* and *fifth* conclusions are the only ones having any real bearing on the case, and each of these is demoralized by *petitio principii.* It is not proved that contagious poisons are not soluble, and so far as cholera is concerned, it is not proved that its poison does more than set in action changes in the textures, which unquestionably do require elimination to prevent fatal result. In the fifth conclusion there is simply a denial of the primary law of osmosis. Briefly, this whole onslaught upon the physiological doctrine of cholera has its analogue in the efforts of narrow-minded divines to do away with the primal truths of geology and natural science. The too zealous religionist is a little in danger of becoming a bigot. The too enthusiastic microscopist is somewhat in danger of becoming microscopic, and a vitalist of the Stahl and Paine stamp. *Ecce signum!*

*Hydrophobia* is the latest sensation in this city. At least one unmistakable and fatal case has occurred, and the usual variety asserted to be such. Death has held high carnival among the canine population, although innumerable curs of high and low degree still (but unquiet) haunt the streets and alleys of the town. The Board of Health is yet in the milky way. Notwithstanding the constellation of talent it embodies, it has not yet cut its *canine* teeth.

Opinions clash and methods jar, whilst BROMIDE OF POTASSIUM is quoted as specific for the semi-quadrupedal disease. A case of cure by its wonderful influence is chronicled by an Indiana paper, and the report is caught up and scattered far and wide by the daily press of the metropolis. Meanwhile a dozen doctors strongly assert, in secular print, "they always knew it" as just the thing for Hydrophobia. Thus Bromide of Potassium ascends to the pedestal recently vacated by Chlorate of Potash and its more or less popularly noted predecessors.

*The Dry Tongue* of fevers so often believed to denote very grave conditions of the general system—"locking up of the secretions," *et alii*, our learned and excellent colleague Prof. FREER is wont to remark in very many, if not a majority of, instances denotes little more than occlusion of the nostrils. Syringing out these latter gently and carefully will, in an unexpected number of cases, rapidly convert the dry and even dark, red tongue to one sufficiently moist and pale to preclude the necessity of resorting to "alteratives," even the aromatic, but nevertheless badly smelling, product of the North Carolina pine. Patients are congratulated on the probably happy results of this suggestion.

*Light for the Endoscope.*—Desormeaux, whose valuable "Lessons of the Endoscope" the JOURNAL is having translated for its readers, will recognize with pleasure the suggestions of our friend E. Andrews, M.D., (formerly of the University of Michigan, at Ann Arbor,) which is that the illuminating rays be derived from the ignited Magnesium wire. We are indebted to the pages of a contemporary (edited by a gentleman formerly from Binghampton in the State of New York—probably not yet forgotten in that region,) for the information.

*Apropos* of the *Endoscope.* It is unnecessary to be at the expense or trouble of procuring the Parisian instrument, which is at once complicated, inconvenient, and costly. The endoscope tube fitted to the reflecting auroscope, and lighted by the ignited Magnesium wire, is much better, and the entire apparatus need not cost more than fifteen or twenty dollars. Messrs. Bliss & Sharp, of 144 Lake Street, this city, can furnish the parts, and our advertisers, Messrs. Tolle & Degenhardt of 125 LaSalle Street, can adjust them to order. This is a new field of diagnosis and is well worth working.

*Belladonna—Scarlatina.*—Bouchut the eminent writer on Diseases of Children, noticing the use of Belladonna as a prophylactic of Scarlatina, writes:

"The greatest objection—but it is not one in our opinion—the greatest objection which can be made to this therapeutic means is, that it does not fulfil the purpose proposed, that in a word, it is useless."

This strikes the Journal as eminently *a la Francaise.*

